# The Study of the Protection Mechanism of Calycosin-7-*O*-β-d-Glucoside Against Oxygen–Glucose Deprivation/Reperfusion in HT22 Cells Based on Non-Targeted Metabolomics and Network Analysis

**DOI:** 10.3390/molecules30030549

**Published:** 2025-01-25

**Authors:** Die Pei, Jieyi Huang, Shanru Chen, Qihui Deng, Cong Nie, Lixia Zhu, Yingfeng Zhang

**Affiliations:** 1 College of Chinese Materia Medica, Guangzhou University of Chinese Medicine, Guangzhou 510000, China; peidie678@163.com (D.P.); hjy20221120311@163.com (J.H.); csr13415141801@163.com (S.C.); dengqihui2023@163.com (Q.D.); nc18379557145@163.com (C.N.); 2 Zhujiang Hospital of Southern Medical University, Guangzhou 510000, China

**Keywords:** cell non-targeted metabolomics, calycosin-7-*O*-β-d-glucoside, oxygen–glucose deprivation/reperfusion

## Abstract

The cell non-targeted metabolomics technique was used to investigate the potential mechanism of Caly-cosin-7-*O*-β-d-glucoside (CAG) against cell oxygen–glucose deprivation/reperfusion (OGD/R). The OGD/R-injured HT22 cell model was constructed. The cells were divided into control, OGD/R, Edaravone (EDA), CAG-L, CAG-M, and CAG-H groups. The protective effect of CAG on OGD/R-injured nerve cells and its potential mechanism was investigated by detecting ROS levels, apoptosis rate, glutamic acid (Glu), γ-aminobutyric acid (GABA), nitric oxide (NO), and combining with cell non-targeted metabolomics. The results showed that after OGD/R, ROS levels, apoptosis rate, Glu and NO concentrations were significantly increased, while the concentrations of GABA were decreased considerably, which improved in a dose-dependent manner after CAG intervention. Cell non-targeted metabolomics results showed that CAG can dramatically improve the metabolomic characteristics of OGD/R-injured HT22 cells. Through bioinformatics analysis and molecular docking, it was found that purine metabolism may be an important pathway for CAG to treat OGD/R injury, and key proteins screened may be important targets for improving OGD/R injury. Therefore, CAG may protect OGD/R-injured HT22 cells by inhibiting apoptosis and oxidative stress, improving energy supply and the metabolomic characteristics of OGD/R-injured HT22 cells by regulating purine metabolism.

## 1. Introduction

Stroke is the world’s second most prominent cause of death and the primary cause of disability, with fatalities increasing year by year [1]. The incidence of ischemic stroke accounts for 85% of the total incidence of stroke, and the age of onset tends to be younger and younger, and the rates of ischemic stroke are increasing in young people [2,3]. The evolution of ischemic stroke involves a series of pathophysiological links such as excitotoxicity, mitochondrial dysfunction, oxidative stress, neuroinflammatory response and apoptosis [4]. In response to these links, signal transduction pathways in ischemic stroke can be classified into several major categories: cell cycle regulation, mitochondrial ATP energy metabolism, and neuroinflammation [5]. Catheter reperfusion and intravenous thrombolysis with tissue plasminogen activator (tPA) are often used clinically to reduce tissue damage in patients with cerebral ischemia, but the inability of many patients to seek or obtain this treatment promptly has led to severe brain damage and disability, thus, the current state of stroke treatment is still faced with limitations, and the growing urgency of developing highly effective neuroprotective agents for acute ischemic stroke. Neuroprotectants are drugs or treatments that protect nerve cells from further damage and help restore or maintain normal neurological function. Their action mechanism involves various aspects such as reducing oxidative stress in nerve cells, inhibiting inflammatory responses, improving energy metabolism, and enhancing cellular self-repair capacity. The core focus of developing neuroprotective agents is to mitigate potential harm during recanalization therapy, widen the therapeutic time window, and further optimize the long-term functional rehabilitation outcomes for patients [6]. The main pathological feature of ischemic stroke is the occlusion of blood vessels in the brain leading to local ischemia, hypoxia, and even necrosis of cerebral neuronal cells [7]. Oxygen–glucose deprivation/reperfusion (OGD/R) is an experimental method to simulate the pathological process of ischemic stroke in vitro. The OGD/R model simulates the pathogenesis of ischemic stroke by artificially removing oxygen and glucose from cell culture in vitro [8]; researchers can observe and analyze the effects of OGD/R on cells at the cellular or molecular level, including apoptosis, calcium overload, oxidative stress, increased excitatory amino acids, and impaired energy metabolism [9], which provides great convenience for in vitro studies of stroke, and the OGD/R model reproduces and accurately mimics the pathological and physiological processes of ischemic stroke, so it is an ideal model for in vitro study of ischemic stroke. Studies have found that Calycosin-7-*O*-β-d-glucoside (CAG) has a neovascularization effect, which can activate the release of the endothelial progenitor cell (EPC) mobilization factors in the organism and induce the participation of the EPCs in the process of regenerative and repairing of the blood vessels after cerebral ischemia [10]. CAG can also reduce neuronal cell damage, improve neurological function in ischemic stroke rats, increase the viability of hippocampal neuronal cells injured by OGD/R, and protect against ischemic brain injury by regulating the silent information regulator 1 (SIRT1) pathway [11,12].

Metabolomics, as an emerging systems biology technology, can precisely capture the molecular interactions between biomarkers and active compounds produced by biological systems faced with pathological stimuli and comprehensively study them to characterize the systemic therapeutic effects of drugs [13]. The analysis of in vitro experiments is more controllable than in vivo experimental analyses, and metabolomics techniques can detect and characterize endogenous biochemical reaction products and reveal metabolic pathways and biological processes within living cells, thus cell metabolomics is gradually developing as a valuable resource to be exploited [14]. Cell metabolomics is a branch of metabolomics that focuses on the quantitative and qualitative analysis of all small molecular weight metabolites of a particular cell at a specific physiological period, with the goal of information modeling and systems integration to reveal cellular metabolic networks and metabolic changes [15,16]. Cell metabolomics can accurately describe the real-time biochemical responses of cells and biochemical characterization of cells in different cell populations, and it can help researchers discover the special characteristics of cell populations in the field of medicine, which has achieved many significant results in the early diagnosis of diseases, drug target discovery, and the study of disease mechanisms [17].

The present study intends to screen the potential biomarkers of CAG intervening in OGD/R-injured HT22 cells at the cellular level based on the cell non-targeted metabolomics technique by using ultra-performance liquid chromatography–quadrupole-time of flight-mass spectrometry (UPLC-Q/TOF-MS), to explore the neuroprotective effects of CAG from the perspective of metabolic network regulation, and to provide a research basis for the treatment of ischemic stroke.

## 2. Results

### 2.1. Effects of Different Oxygen–Glucose Deprivation (OGD) Times on the Viability of HT22 Cells

The results of OGD/R modeling time screening are shown in Figure S2 of Supplementary Materials. Compared with the control group, the cell viability decreased to 52% when oxygen and glucose were deprived for 10 h. Therefore, 10 h of OGD was utilized as the modeling time.

### 2.2. Non-Toxic Dose Range of CAG and Edaravone (EDA)

**Figure 1:**
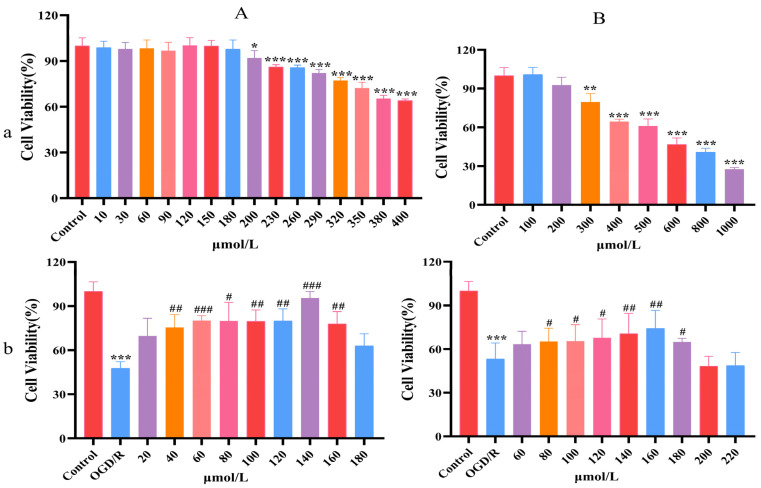
Confirmation of the non-toxic dose range and dosage of CAG and EDA. (**A**) CAG; (**B**) EDA; (**a**) non-toxic dose range; (**b**) dosage confirmation. (mean ± SD, *n* = 6) (* *p* < 0.05, ** *p* < 0.01, *** *p* < 0.001, compared with the control group; ^#^ *p* < 0.05, ^##^ *p* < 0.01, ^###^ *p* < 0.001, compared with the OGD/R group).

As can be seen from Figure 1A(a),B(a), the cell survival rate decreased significantly when the CAG concentration was 200 μmol/L (*p* < 0.05). The IC50 of CAG is about 480 μmol/L (95% confidence interval: 458.9 μmol/L to 506.0 μmol/L). There was a noticeable reduction in the cell survival rate at 300 μmol/L of EDA (*p* < 0.01). According to the non-toxicity range of CAG, CAG concentration was set as 20, 40, 60, 80, 100, 120, 140, and 180 μmol/L for the follow-up study. EDA concentration was set as 60, 80, 100, 120, 140, 160, 180, 200, and 220 μmol/L for the follow-up study.

### 2.3. Confirm the Dosage of CAG and EDA

As shown in Figure 1A(b),B(b), there was the highest survival rate in OGD/R-injured HT22 cells when the CAG concentration was 140 μmol/L. Therefore, 35, 70, and 140 μmol/L CAG amounts were selected as the low, medium, and high doses. The survival rate of HT22 cells after the intervention of 160 μmol/L EDA in OGD/R injury was the highest. According to the results, 160 μmol/L EDA was utilized as the positive control for follow-up experiments.

### 2.4. The Effect of CAG on the Viability of OGD/R-Injured HT22 Cells Was Studied by the CCK-8 Method

**Figure 2:**
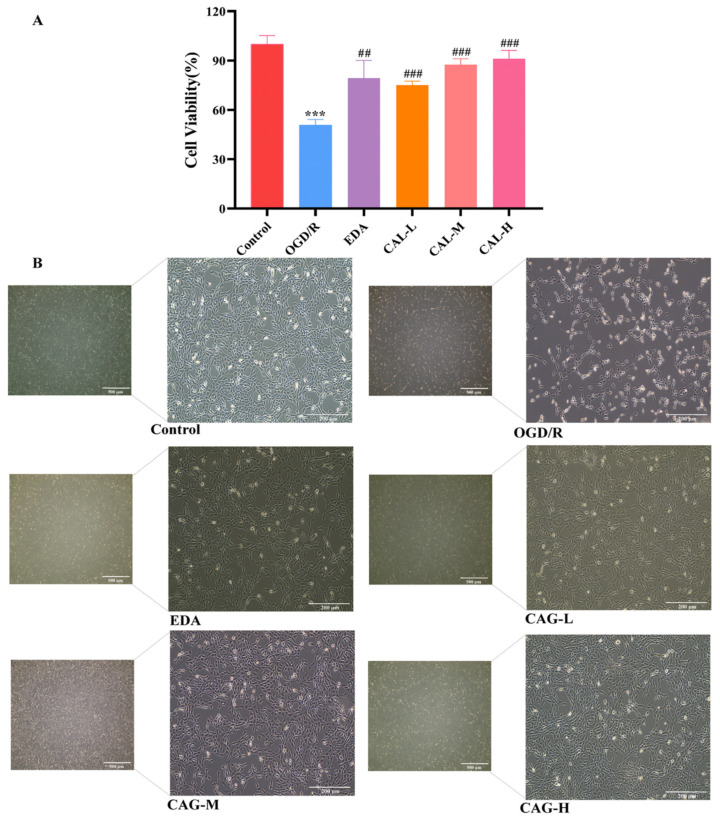
Cell viability and cell morphology. (**A**) The effect of CAG on OGD/R-injured HT22 cell viability was detected by the CCK-8 method. (**B**) Cell morphology (the scale bar for low magnification and high magnification is 500 μm and 200 μm, in that order). (*** *p* < 0.001, compared with the control group; ^##^ *p* < 0.01, ^###^ *p* < 0.001, compared with the OGD/R group) (mean ± SD, *n* = 6).

As shown in Figure 2A, HT22 cell viability decreased significantly after modeling (*p* < 0.001). Compared to the OGD/R group, the cell viability of HT22 cells injured by OGD/R was significantly enhanced in CAG low-dose (CAG-L), CAG medium-dose (CAG-M), and CAG high-dose (CAG-H) groups (*p* < 0.001), and the cell protection effect was dose dependent. Figure 2B depicts that HT22 cells in the control group had smooth morphology, clear contour, nerve antennae, and good adhesion to the culture dish. In the OGD/R group, HT22 cells became round, intercellular connections decreased, some cell clusters sparsely fell off, cells shrunk, cell cavities narrowed, and there was poor cell adhesion. After CAG and EDA intervention, the cell morphology was significantly improved, the nerve antennae were extended, and the cell adhesion was good.

### 2.5. The Effect of CAG on ROS Levels in OGD/R-Injured HT22 Cells Was Detected by Flow Cytometry

**Figure 3:**
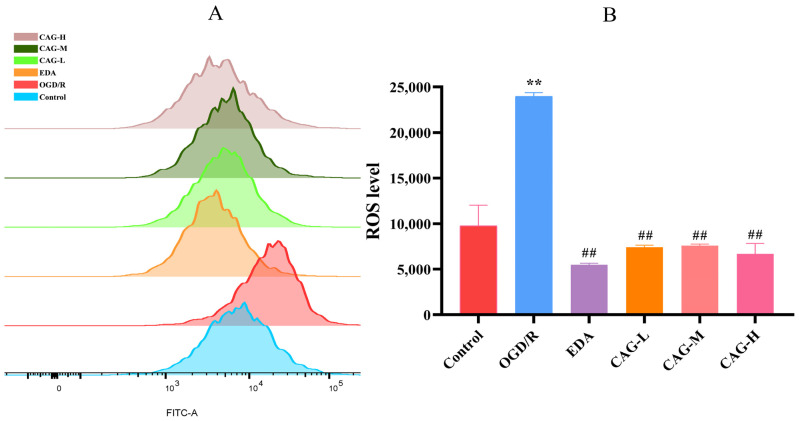
DCFH-DA probe and flow cytometry were used to investigate the changes in ROS levels. (**A**) Histogram of ROS fluorescence intensities superimposed on each group of cells. (**B**) ROS fluorescence intensity (fluorescence intensity is proportional to ROS level). (** *p* < 0.01, compared with the control group; ^##^ *p* < 0.01, compared with the OGD/R group) (mean ± SD, *n* = 6).

DCFH-DA is a probe that can freely cross the cell membrane and is hydrolyzed by esterase to produce DCFH in the cell. DCFH is then oxidized by ROS to produce DCF with fluorescence whose fluorescence intensity is proportional to intracellular ROS levels. Therefore, the ROS level can be analyzed by using flow cytometry to detect the fluorescence signal intensity in living cells. The most significant characteristic of oxidative stress is the excessive accumulation of ROS. The results proved that ROS levels of HT22 cells were considerably elevated after OGD/R injury compared with the control group (*p* < 0.01). Compared to the OGD/R group, CAG could significantly reduce ROS levels (*p* < 0.01). The results showed that CAG could effectively reduce the increase in the peroxidation level of nerve cells caused by cerebral ischemia, thus alleviating the oxidative stress response of neurons (see Figure 3).

### 2.6. The Effect of CAG on Apoptosis of OGD/R-Injured HT22 Cells Was Detected by Flow Cytometry

**Figure 4:**
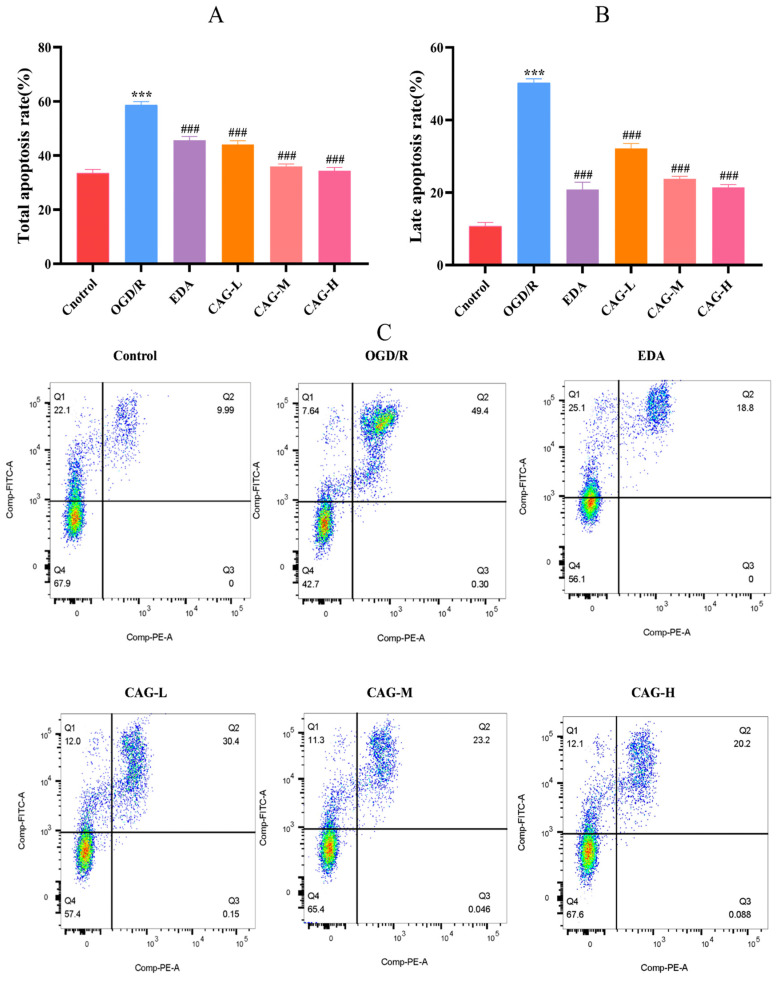
Cell apoptosis. (**A**) Total apoptosis rate; (**B**) late apoptosis rate; (**C**) apoptosis fluorescence diagram (mean ± SD, *n* = 6) (Q4 means living cell; Q1 means early apoptotic cell; Q2 means late with apoptotic cell; Q3 means bare nucleus cells). (*** *p* < 0.001, compared with the control group; ^###^ *p* < 0.001, compared with the OGD/R group).

Figure 4 showed that in comparison to the control group, the apoptosis rate of HT22 cells in the OGD/R group was significantly increased (*p* < 0.001). Compared with the OGD/R group, HT22 cells’ apoptosis rate significantly decreased after CAG treatment (*p* < 0.001). With the increase in CAG concentration, the apoptosis rate decreased in a dose-dependent manner. After OGD/R injury, the late apoptosis rate of the cells increased most obviously (*p* < 0.001), and bare nucleus cells appeared. After EDA administration, the late apoptosis rate was significantly decreased (*p* < 0.001), but the early apoptosis rate was still high. Unlike the EDA group, CAG administration showed a downward trend in both early and late apoptotic rates, and the decrease in late apoptosis rate was more significant.

### 2.7. Determination of Glu, GABA, and NO Concentration

**Figure 5:**
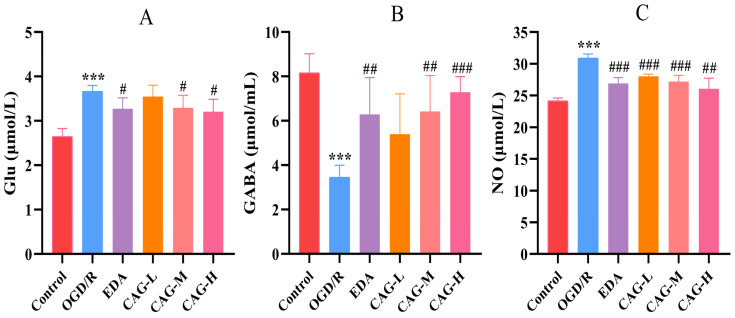
Concentration of Glu, GABA, and NO. (**A**) Glu; (**B**) GABA; (**C**) NO. (mean ± SD, *n* = 6). (*** *p* < 0.001, compared with the control group; ^#^ *p* < 0.05, ^##^ *p* < 0.01, ^###^ *p* < 0.001, compared with the OGD/R group).

The results of Glu, GABA, and NO concentration are shown in Figure 5. Compared with the control group, the concentration of Glu and NO was significantly increased, and GABA concentration was significantly decreased in the OGD/R group (*p* < 0.001). Compared with the OGD/R group, the concentration of Glu in EDA, CAG-M, and CAG-H groups was significantly decreased (*p* < 0.05), the concentration of NO in cell supernatants in EDA, CAG-L, CAG-M, and CAG-H groups was significantly decreased (*p* < 0.01), and GABA concentration in EDA, CAG-M, and CAG-H groups was increased considerably, which showed obvious dose–effect relationship characteristics (*p* < 0.05).

### 2.8. Cell Non-Targeted Metabolomics Studies

#### 2.8.1. Multivariate Statistics of Cell Non-Targeted Metabolomics and Metabolic Profile Analysis

**Figure 6:**
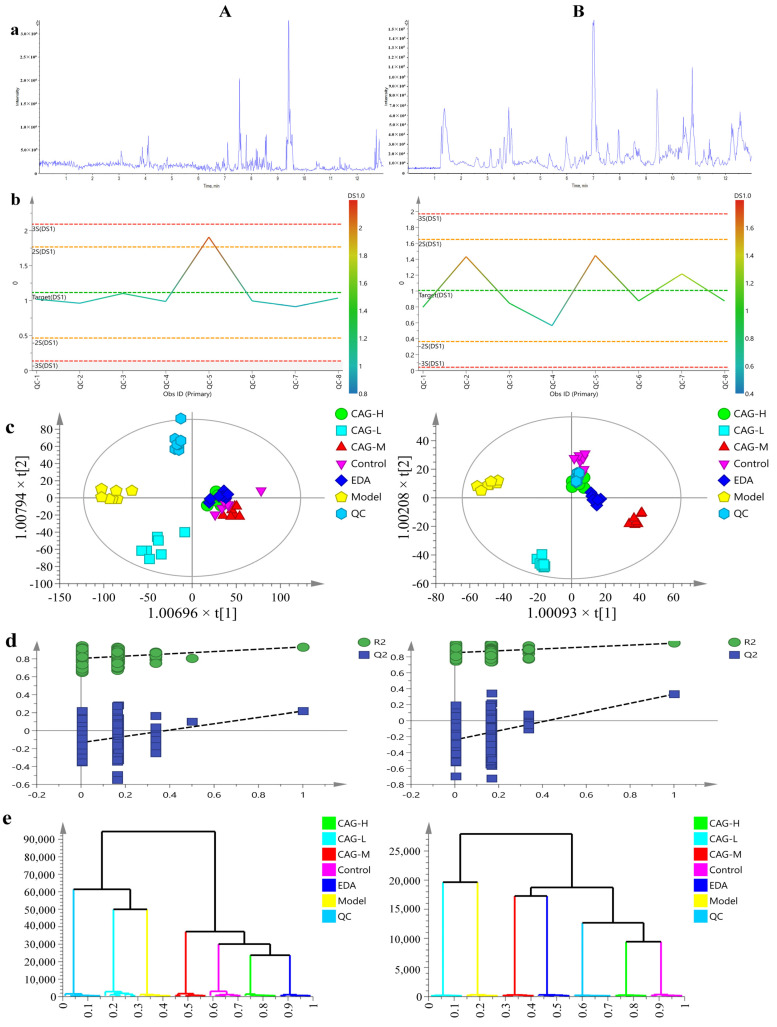
Multivariate statistics of cell metabolomics and metabolic profile analysis. (**A**) Positive ion mode; (**B**) negative ion mode; (**a**) BPC diagram (take QC as an example); (**b**) QC sample control diagrams; (**c**) OPLS-DA score scatter plot; (**d**) 200 iteration permutation test diagram; (**e**) cluster analysis plot.

The fluctuation of quality control (QC) samples in positive and negative ions was within ± 3SD, indicating that the fluctuation of the instrument was small, which met the technical requirements of metabolomics analysis (Figure 6b). The relative distribution of samples in the PCA plots in positive and negative ion modes is similar in the 95% confidence interval. The QC samples are highly coincident and located near the center, indicating that the entire operation process has good controllability and reliability. In PCA score plots, EDA, CAG-H, and CAG-M groups were highly overlapping, indicating small changes in different metabolites between groups, with highly identical trends and similar metabolomic characteristics. Samples from the OGD/R group were far away from the control group and the drug intervention group, indicating significant changes in metabolites of HT22 cells after modeling. In general, the separation trend between the OGD/R group and other groups is more obvious in the positive ion mode (See Figure S3). The results of the OPLS-DA model fitting parameters and cross-validation analysis of variance (CV-ANOVA) are shown in Table 1. Theoretically, the closer the value of R2 (cum) and Q2 (cum) is to 1, the better the model, while the lower the value, the worse the fitting precision of the model; R2 (cum) and Q2 (cum) should be higher than 0.5 [18]. The R2 (cum) > 0.9 and Q2 (cum) > 0.5 of the model in this study indicate that the model is completely acceptable (Table 1). The cell metabolomics data in positive and negative ion modes were processed by orthogonal partial least squares discriminant analysis (OPLS-DA), the score scatter plot of the OPLS-DA model is shown in Figure 6c. QC samples are relatively clustered, indicating high instrument stability. Samples in each group are clustered, with small dispersion, and the metabolic profile is significantly different between the control group and the OGD/R group. This showed that HT22 cells underwent obvious changes after OGD/R. The metabolic profile of the OGD/R group is significantly different from that of the CAG-L, CAG-M, and CAG-H groups, indicating that after CAG intervention, the metabolic profile of OGD/R cells changes significantly, and drugs play a protective role. The difference between the control group and the CAG-H group was very small, indicating that after CAG intervention, the metabolomic characteristics of HT22 cells injured by OGD/R were significantly reversal. The overfitting of the OPLS-DA model was evaluated by 200 iteration permutation tests. As shown in Figure 6d, all blue Q2 values to the left are lower than the original points to the right. The blue regression line of the Q2 points intersects the vertical axis below zero. When R2 > Q2 is in positive ion mode, the intercept of R2 is 0.805 and the intercept of Q2 is −0.133; when R2 > Q2 is in negative ion mode, the intercept of R2 is 0.852 and the intercept of Q2 is −0.24; Q2 is smaller than R2 in positive and negative ion modes. Moreover, the intersection points of the regression line of Q2 and the *Y*-axis are in the negative semi-axis, indicating that the model has good stability, is not over-fitting, and has good prediction ability and reliability.

The SUS plot results are shown in Figure S4. From the figure, it can be seen that the metabolites in control vs. model-EDA vs. model, control vs. model-CAG-H vs. model and EDA vs. model-CAG-H vs. model under positive and negative ion modes were distributed near the 45° lines from the lower left corner to the upper right corner, indicating that the profiles of control, CAG, and EDA metabolites were highly similar after drug intervention.

#### 2.8.2. Differential Metabolite Analysis

**Figure 7:**
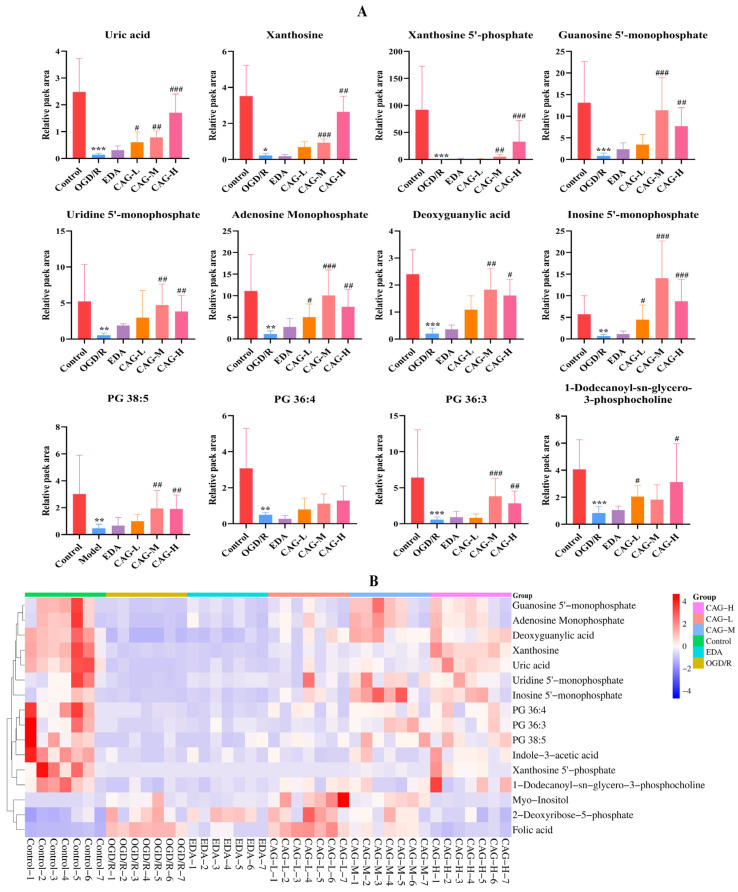
(**A**): Column bar graph of partial differential metabolites (mean ± SD, *n* = 7); (**B**) clustering heatmap of differential metabolite (the change of color scale indicates the trend of the relative expression level; the change of color scale under “Group” indicates different groups). (All metabolites in each group of samples are normalized to 3 × 10^6^ cells). (* *p* < 0.05, ** *p* < 0.01, *** *p* < 0.001, compared with the control group; ^#^ *p* < 0.05, ^##^ *p* < 0.01, ^###^ *p* < 0.001, compared with the OGD/R group).

Under the condition that the established OPLS-DA model fitted well, FC > 2 or < 0.5, VIP > 1 and *p* < 0.05 were used as the criteria, and the S-plot and volcano plots were combined to screen out the potential differential metabolites between the control group and the OGD/R group (Figure S5). Finally, 16 potential differential metabolites were obtained. Combined with Table 2 and Figure 7, the expression levels of metabolites such as Uric acid, Xanthosine, Xanthosine 5′-phosphate (XMP), Guanosine 5′-monophosphate (GMP), Uridine 5′-monophosphate (UMP), Adenosine Monophosphate (AMP), Deoxyguanylic acid (dGMP), Inosine 5′-monophosphate (IMP), and PG 36:3 in the OGD/R group were decreased compared with the control group. After the intervention of CAG and EDA in OGD/R-injured cells, these significantly down-regulated differential metabolites had a significant callback to different degrees and tended to the control group. Figure 7B clustering heatmap is an intuitive visualization method for differential metabolites, which uses gradient colors in specific intervals to intuitively display the relative expression of differential metabolites, with red representing the relative high level of differential metabolite expression and blue representing the relative low level of expression, and the color scale changes indicate the high or low expression level and the trend of change. The change of color scale indicates the trend of the expression level, so the clustering heatmap can more intuitively show the differences between groups and the up-/down-regulation distribution of metabolites (Figure 7B). Through the clustering heatmap and column bar graph, we can find that CAG and EDA have different degrees of significant callback effect on differential metabolites, and tend to be normal. It can be seen that CAG and EDA have good efficacy. The metabolite correlation network can more clearly characterize the correlation between metabolites (Figure S6).

#### 2.8.3. Multivariate Exploratory ROC Analysis Result

The Receiver Operating Characteristic Curve (ROC) is a powerful tool for evaluating differential metabolites to distinguish between normal/pathological conditions and control/model groups. The ROC curve can demonstrate the efficacy of the differential metabolites under different thresholds. The area under the curve (AUC) is the main parameter of the ROC curve, and the closer the value of AUC is to 1, the better the discriminatory ability of the model is. The AUC of all the differential metabolites in this study was greater than 0.9, indicating that they could effectively distinguish between model cells and normal cells. However, a single metabolite often fails to fully reflect the differences between model cells and normal cells. Therefore, this study used multi-indicator ROC analysis to discover the combination of differential metabolites with the smallest number and the strongest discriminatory ability to more comprehensively reveal the differential characteristics between normal cells and model cells. Based on the MetaboAnalyst 6.0 website, the Monte Carlo cross-validation (MCCV) algorithm was used to generate the ROC curves, combined with the linear support vector machine (SVM) classification method and the built-in support vector machine (SVM) feature ranking method was used to rank the result of the multivariate exploratory ROC analysis obtained by balanced subsampling [19]. Using AUC and CI as indicators, the website gives the best differential metabolite groups. Multivariate joint analysis showed that the top 10 most valuable potential biomarkers include uric acid, 2-Deoxyribose-5-phosphate, Xanthosine, Myo-Inositol and so on (See Figure S8A(b)). Uric acid, 2-Deoxyribose-5-phosphate, Xanthosine, Myo-Inositol, and Deoxyguanylic acid may serve as the most valuable potential joint diagnosis indicators for OGD/R cells (See Figure S8A). Multivariate exploratory ROC analysis enhanced the discriminatory efficacy of the model. The optimal differential metabolite combinations found in this study may be helpful for the diagnosis of stroke disease and the evaluation of drug efficacy.

#### 2.8.4. Metabolic Pathway Analysis

The online platform MetaboAnalyst 6.0 was used for the metabolic pathway and enrichment analysis of differential metabolites with significant callback effects by CAG. As shown in Figure S8B(a), the larger the pathway impact value, the larger the node radius. As shown in Figure S8B(b), the darker the color, the smaller the *p*-value obtained by pathway enrichment analysis. Specifically, the shade of red of the dots is based on the *p*-value, the color from lighter to darker, and the *p*-value from larger to smaller, i.e., the darker the color represents the smaller the *p*-value and the higher the significance level. The radius of the nodes of the dots is based on their pathway impact value, from small to large node radius, the impact value is from small to large. Therefore, the node radius and color shades of the dots in the graph together reflect the statistical significance and biological impact of the metabolic pathways in the enrichment analysis. Therefore, in the metabolic pathway diagram, the larger the node radius and the darker the color, the more differential metabolites involved in this pathway, and the more significant role in the overall metabolic profile. The results showed that in the whole metabolic profile, the most significant metabolic pathway was purine metabolism, with *p* < 0.001 and impact value > 0.1, meeting the conditions of *p* < 0.05 and impact value > 0.1 for pathway screening.

#### 2.8.5. Construction and Analysis of Differential Metabolite Bio-Information Network

The differential metabolites that can have a significant callback by CAG were used for biological network construction and analysis, and their potential anti-OGD/R injury targets were explored. MetScape plug-in was used to construct a metabolite–gene network (See Figure S7A), and 138 differential metabolite-related gene targets were obtained. A search of the GenBank with the keyword “Ischemic stroke” yielded 5111 disease gene targets. Forty intersection targets of metabolite-related genes and disease genes were obtained through the Venn diagram (See Figure S7B). KEGG enrichment analysis (Figure S8C(a)) and GO analysis (Figure S8C(b)) were performed on 40 common targets by Cluego plug-in of Cytoscape software (Version 3.9.1). The results of KEGG enrichment analysis showed that histidine metabolism, purine metabolism, pyrimidine metabolism, and ubiquitin-mediated proteolysis are involved in the pathological process of OGD/R. A total of 65 biological processes, including the adenosine metabolic process, AMP metabolic process, GMP metabolic process, and hydrogen peroxide metabolic process, and 17 molecular functions, including oxidoreductase activity, nucleobase-containing compound kinase activity, and nucleotidase activity, were enriched by GO analysis. The 40 common targets obtained by the Venn diagram were imported into the STRING online database, and the processed protein–protein interaction (PPI) data were imported into Cytoscape software (Version 3.9.1) to further beautify and visualize the PPI network (Figure S8D(a)). MCODE plug-in was used to rank the network nodes of the data, and 16 key proteins related to HT22 cells with OGD/R injury were obtained (Figure S8D(b)), among which the top key targets were Adk, Aprt, Entpd1, Pde4d, Enpp3, and Nt5c2, etc.

The online platform MetaboAnalyst 6.0 was used to conduct joint-pathway analysis of the obtained key proteins and differential metabolics that could be significantly reversed by CAG (Figure S8E). Four pathways with *p* < 0.05 and impact value > 0.1 were obtained. These are purine metabolism, pyrimidine metabolism, nicotinate and nicotinamide metabolism, and riboflavin metabolism. The trends of purine metabolism and pyrimidine metabolism are shown in Figure S8F(a),F(b). It is clear from Figure S8F that the differential metabolites identified in this study, including uric acid, Xanthosine, XMP, GMP, UMP, AMP, and dGMP, play a crucial role in key biological processes such as purine signaling pathways. The expression changes of these differential metabolites not only reveal the activity state of the signaling pathway from the level of metabolites but also their regulation during treatment indirectly reflects the effect of drug therapy. By monitoring changes in these metabolites, we can gain a deeper understanding of the mechanisms of drug action and assess the effectiveness of treatment.

#### 2.8.6. Molecular Docking

A target of degree value greater than or equal to twice the median in the MCODE subcluster of the PPI network was selected, and the molecular docking evaluation between CAG and key proteins was performed by the Dockthor molecular docking platform, and the binding force between CAG and target proteins was calculated based on the size of the binding energy. The lower the binding energy, the more stable the interaction between CAG and the target. It is generally believed that binding energy <−4.25 kcal·mol^−1^ indicates that the composition and target can be combined, binding energy <−5.0 kcal·mol^−1^ indicates that the composition and target can be well combined, and binding energy <−7.0 kcal·mol^−1^ indicates that the two have strong binding activity [20]. The results of the docking interaction model between CAG and key proteins are shown in Figure S9. As shown in Figure S9A, 14 key proteins can bind with CAG, among which 13 key proteins are well bound with CAG (binding energy < −5.0 kcal·mol^−1^). Eight key proteins, including Adk, Aprt, Entpd1, Pde4d, Pde4a, Pde5a, Pde3a, and Pde11a, had strong binding activity with CAG (binding energy < −7.0 kcal·mol^−1^). The results suggest that CAG may improve the metabolomics of profile OGD/R-injured HT22 cells, proteins such as Adk, Aprt, Entpd1, and Pde4d may be the action targets.

## 3. Discussion

CAG, the main active ingredient in Astragali Radix, a very commonly used and important traditional Chinese medicine with a tonifying Qi effect, is a plant-derived monomer with an estrogen-like effect. CAG is capable of exerting significant anti-cardiovascular disease activity. CAG shows remarkable antioxidant capacity, which can effectively remove free radicals and thus alleviate the damage caused by oxidative stress. It is reported that CAG can activate the PI3K/Akt signaling pathway as a promising cardioprotective substance, which helps to inhibit the activity of oxidative stress and pro-apoptotic factors [21]. In addition, it can also promote the secretion of IL-10 and activate the JAK2/STAT3 signaling pathway, showing pharmacological effects in alleviating myocardial ischemia/reperfusion injury [22].

Comprehending the physiological and pathological process of cerebral ischemia relies on exploiting animal models of ischemic stroke. However, notable distinctions linger between patients with stroke and animal models of cerebral ischemia. For example, notable distinctions in blood–brain barrier (BBB) function, excitotoxicity, and inflammatory responses were found between rats and humans [23,24]; therefore, in vitro experimental research methods can be used to provide an initial rapid assessment and as a complement to animal model experiments [25]. Once ischemic stroke occurs, cerebrovascular blockage and other causes will lead to local interruption of blood flow to the brain tissue, resulting in a lack of essential oxygen and glucose to the neuronal cells in the affected area. Since the oxygen consumption of brain tissue is much higher than that of other tissues, when a prolonged or permanent blockage occurs in one of the cerebral vascular branches, it leads to irreversible neuronal necrosis [26,27]. OGD/R can simulate the pathological changes of hypoxia and glucose deficiency in ischemic brain tissue at the cellular level, and the cell experimental method for studying the mechanism of ischemic stroke allows for possible searching of neuroprotective strategies. The OGD/R cell model has been widely used to simulate the complex pathological cascade triggered by cerebral ischemia in vivo. Cell metabolomics allows for a comprehensive evaluation of metabolites, providing information that most closely approximates the phenotype of a biological system, and has great potential value in a variety of fields, including toxicology, drug testing, cell culture monitoring, pharmaceutical research, and biopharmaceutical production [28]. As reported by other scholars, CAG has been shown to dramatically decrease BBB permeability and infarct volume following cerebral ischemia/reperfusion injury in rats, and it protects tight junction proteins from degradation by inhibiting MMP-9/2 activity, thereby maintaining BBB integrity. In addition, CAG reduced NO production and inhibited cav-1 down-regulation and MMPs activation. Cell experiments further confirmed that CAG was able to eliminate NO and effectively inhibit MMP-9/2 activation, thereby attenuating the damage and death of microvascular endothelial cells under OGD. Thus, CAG demonstrated multi-pathway antioxidant and neuroprotective effects in ischemia/reperfusion injury [29]. In summary, the cerebral ischemia simulation system of HT22 cells was established by the OGD/R method in this study, and the improvement mechanism of CAG on OGD/R-induced neuronal injury and its potential targets were further explored using oxidative stress analysis, apoptosis detection, and cell non-targeted metabolomics studies, providing a scientific basis for CAG treatment of ischemic stroke.

The phenomenon of cerebral ischemia/reperfusion can induce an imbalance between the generation and clearance mechanism of ROS, leading to oxidative stress [30]. With the deepening of research, more and more scientific evidence has proved that ROS plays a central role in the OGD/R process, and its expression level is closely related to the pathological course of ischemic stroke. When ROS production is excessive or the clearance system is inhibited, intracellular ROS accumulation leads to an oxidative stress response, which promotes apoptosis through various mechanisms such as affecting mitochondrial function, DNA damage repair, and activation of specific signaling pathways [31]. CAG has been depicted to decrease oxidative stress and apoptosis, enhance Bcl-2 expression in cells, decrease Bax expression, and reduce ROS generation caused by OGD/R via the SIRT1/FOXO1/PGC-1α signaling pathway, and thus prevent OGD/R-induced hippocampal cell damage [32]. This study showed that after OGD/R injury of HT22 cells, the intracellular ROS level and apoptosis rate increased significantly, and cell viability decreased significantly. After the intervention of CAG on the injured cells, the intracellular ROS level and apoptosis rate decreased significantly, and the cell vitality tended to the normal state. It is worth noting that the therapeutic effects of CAG also exhibit a dose-dependent property.

Purine metabolism includes the synthesis and decomposition of purine. Its main role is to participate in energy metabolism, provide energy for cell activities, regulate cell signal transmission, and maintain uric acid balance in the body [33]. Purine is also an important part of DNA and RNA. All brain cells produce and release purines. Purine signaling pathways mainly include adenosine triphosphate (ATP), adenosine diphosphate (ADP), adenosine monophosphate (AMP), and adenosine (ADO), which act on purinergic receptors, prevent thrombosis, and inhibit inflammation, and are potential therapeutic targets for ischemic stroke [34]. In addition, uric acid, a product of purine metabolism, is also one of the most important antioxidants in blood and is a potential neuroprotective substance in vascular recanalization therapy. High serum uric acid level is a good predictor of outcomes in patients with acute ischemic stroke, and the change in its level is closely related to the prognosis of stroke [35]. This study has established a rigorous and scientific methodology for metabolomics research, strictly adhering to standardized protocols to systematically control key aspects such as sample preparation, experimental analysis, quality control, metabolite identification, and data preprocessing [36]. The focus of the study was to evaluate the impact of CAG on the metabolomics of OGD/R-injured neuronal cells based on the changes in levels of differential metabolites. The threshold criteria of differential metabolites for non-targeted metabolomics are generally fold change (FC) > 2 or < 0.5, *p* < 0.05, VIP > 1, and if these three criteria are met at the same time, they are considered differential metabolites [37,38]. This approach adheres to the technical standards for differential metabolite screening in metabolomics and is a mainstream method [39]. Therefore, this study integrated the above three criteria to construct new volcano plots with three dimensions, including FC, *p*-value, and VIP. In combination with the S-plot of the OPLS-DA model, we comprehensively, objectively, and rigorously screened differential metabolites. The results showed that when neuron cells were injured by OGD/R, compared to the control group, the expression levels of intracellular uric acid, Xanthosine, Xanthosine 5′-phosphate, Guanosine 5′-monophosphate, Uridine 5′-monophosphate, and Adenosine are significantly decreased, which leads to the disturbance of purine metabolism pathway, and then induces serious insufficiency or even complete interruption of energy supply. However, after the intervention of CAG, we observed that the levels of the metabolites in the purine metabolism pathways were effectively reversed. Specifically, the content of these metabolites gradually returned to near-normal levels, CAG effectively ameliorated the disorder of purine metabolism pathways caused by OGD/R injury. This improvement led to restoring energy supply, providing the necessary energy support for neurons, and ultimately playing a significant neuroprotective role. Exploring metabolites with combined diagnostic value may help to improve the accuracy and reliability of the diagnosis of ischemic stroke. This study found that uric acid, 2-Deoxyribose-5-phosphate, Xanthosine, Myo-Inositol, and Deoxyguanylic acid are differential metabolites that have been screened for good combined diagnostic value. The combined detection of uric acid, an end product of purine metabolism, and 2-Deoxyribose-5-phosphate, a key intermediate in DNA synthesis and repair, and Xanthosine, Myo-Inositol, and Deoxyguanylic acid may be important for the diagnosis of ischemic stroke. In summary, purine metabolism may be integral to the pathogenesis, neuroprotection, and prognosis assessment of stroke. Uric acid, as an important product of purine metabolism, may be closely related to the prognosis of stroke. The purine signaling pathway provides a new target for treating stroke, which can be further studied in the future. This study not only revealed the potential of CAG in improving the metabolomics characteristics of neuron cells after OGD/R injury but also provided a powerful experimental scheme and basis for further exploration of neuroprotective strategies.

In the central nervous system, ATP can be stored in vesicles alone or with neurotransmitters and released by neurons via exocytosis or as a co-transmitter of Glu or GABA, in excitatory Glu synapses and GABA synapses [40]. In neurons, the amount of ATP in synaptic vesicles is comparable to GABA and Glu, suggesting that this nucleotide plays an important role in synaptic function and is strongly related to Glu and GABA [41]. In the process of neuronal cell injury caused by cerebral ischemia, rapid depletion of oxygen supply and energy can lead to active oxidative stress, inflammation, damage to the blood–brain barrier, release of excitatory amino acids, etc. Purine metabolism not only provides precursors and energy for synthesizing neurotransmitters but also directly participates in intracellular signal transmission through its metabolites (such as ATP). The disruption of purine metabolism can greatly interfere with the supply of energy to the brain, resulting in impaired reuptake of neurotransmitters such as Glu and GABA. Substances such as Glu, GABA, and NO cooperate with ATP to participate in intercellular energy metabolism and signal transduction, while purine metabolism is involved in energy metabolism and provides energy for cell life activities. Therefore, with the change fluctuation of intermediate products of purine metabolism, the level of neurotransmitters and signal molecules also changes. The detection of GABA, Glu, and NO levels can indirectly reflect the balance of purine metabolism and the status and changes of stroke patients. A metabolic transformation relationship between Glu and GABA is essential to maintaining the relative balance of excitatory and inhibitory systems in the brain [42]. This study showed that after OGD/R injury, the release of Glu in the cells increased significantly, which triggered the overactivation of Glu receptors, resulting in excitatory toxicity of neuron cells. GABA release is reduced, resulting in an imbalance in the Glu to GABA ratio, ultimately leading to neuronal damage. In summary, the increased Glu concentration and decreased GABA concentration in neuron cells after OGD/R injury reflect the severity of cell injury to a certain extent, which are important biomarkers for evaluating the condition and prognosis after stroke.

Adenylate Kinase (Adk) is a crucial enzyme closely associated with intracellular energy metabolism and signal transduction. It maintains adenine nucleotide homeostasis by catalyzing the interconversion between ATP and AMP. Adk also regulates cell cycle progression and mitochondrial ATP energy transfer, allocating energy during cellular processes [43]. During ischemic stroke, cerebral blood flow disruption leads to hypoxia and glucose deprivation in brain tissue, disrupting cellular energy metabolism. Adk may participate in cellular responses to ischemia by modulating ATP and ADP levels, thereby regulating cellular energy metabolism [44]. Furthermore, Adk is involved in apoptosis, necrosis, and autophagy, exerting an anti-apoptotic effect by influencing the levels of apoptosis-related proteins [45]; these processes are pivotal in the onset and progression of ischemic stroke. Our studies suggest that Adk may be a significant target for CAG anti-ischemic stroke, playing an important role in energy metabolism and cell signal transduction after stroke, and may be related to pathophysiological processes such as apoptosis. Adenine phosphoribosyl transferase (Aprt) acts on the catabolic pathway of purines, which can convert adenine to hypoxanthine and promote the recycling of nucleotides. In addition, the purine salvage pathway in plants is mainly catalyzed by Aprt and Adk. This process not only contributes to the production of adenosine nucleotides at the energy level but is also essential for the removal of purine bases and nucleosides that may inhibit metabolic flow. Therefore, Aprt and Adk play an important role in the process of purine metabolism [46]. Ectonucleoside triphosphate diphosphohydrolase 1 (Entpd1), a membrane-binding enzyme, catalyzes the hydrolysis of ATP and ADP in the extracellular environment to generate AMP, which in turn affects extracellular adenosine levels [47]. Adenosine is an important neuroregulatory molecule that can regulate vascular tension, inflammatory response, and neuroprotection by binding to its receptors [48]. Studies have shown that inhibiting the activity of Entpd1 can promote the recovery of neural function in the mouse model of ischemic stroke, because inhibiting Entpd1 reduces the production of extracellular adenosine, thus alleviating the inhibitory effect of adenosine on neural function [49]. Therefore, Entpd1 may affect the recovery of nerve function and immune response by regulating the concentration of adenosine in ischemic stroke, and inhibiting its activity may have potential positive effects on the treatment of stroke. In this paper, through the study of cell metabolomics, the potential key targets of CAG against OGD/R injury were revealed, and the science of related target proteins was initially verified by molecular docking technology. According to the literature review, the core proteins identified, such as Adk, Aprt, Entpd1, and Pde4d, are closely related to the pathogenesis and treatment of ischemic stroke, opening a new path and providing solid theoretical support for the subsequent exploration of pharmacodynamic mechanisms. We have simulated the docking of EDA and Adk, and the results show that the binding sites and energies of EDA and Adk are different from those of CAG-Adk, as shown in Figure S10 for specific simulation results. As can be seen from the visual results of molecular docking between CAG and Adk (Figure S9), the binding energy of CAG and Adk is −8.279 kcal·mol^−1^, and the binding energy of EDA and Adk is −6.933 kcal·mol^−1^. The binding energy of EDA and Adk is weaker than that of CAG. The binding sites of CAG and Adk are LYS97 and GLU98, and the binding sites of EDA and Adk are LYS200; this suggests that the pharmacodynamic mechanisms of CAG and EDA may be inconsistent. In summary, the use of cell metabolomics to study the key target proteins in the treatment of ischemic stroke can not only clarify the core role of drugs in the neuroprotective mechanism and the regulation of inflammatory response but also provide a scientific basis and theoretical support for exploring new therapeutic approaches such as specific receptor inhibitors and antagonists, regulating enzyme activity.

In vitro ischemia models furnish an effective means to support metabolic and cellular functions in ischemic pathophysiological conditions, despite certain variations between in vitro systems and animal models. In this study, we integrated a variety of bioinformatics tools and databases to reveal the molecular mechanism of CAG protection against OGD/R-induced injured HT22 cells from the metabolite level to the gene and protein level. The research first focuses on finding differential metabolites, then tracing the signaling pathway and identifying the target protein. By analyzing the upstream and downstream relationships between large molecules such as proteins and small molecules such as metabolites, we can find out pathways that affect metabolic changes, which helps to discover possible targets and enhances the depth of research. In this study, the OGD/R cell model was used to explore the pharmacodynamic mechanism of CAG to improve ischemic stroke, and it was found that CAG had a good protective effect on HT22 cells injured by OGD/R, and its protective effect might be achieved by alleviating cell oxidative stress, reducing the cell apoptosis rate, and restoring energy supply. Metabolomics studies have found that CAG can significantly improve purine metabolism disorders caused by OGD/R injury, restore energy supply, and have neuroprotective effects.

## 4. Materials and Methods

### 4.1. Materials

Triple TOF 5600+ Time-of-Flight Mass Spectrometer (BN22071312, AB Sciex Pte. Ltd., Framingham, MA, USA); LC-30AD Ultra-High Performance Liquid Chromatography (Shimadzu, Kyoto, Japan; configure the SIL-30AC injector); DxP Athena B4-R2 Flow Cytometer (Cytek Biosciences, Fremont, CA, USA); Full-Wavelength Enzyme Labeling Instrument (ThermoFisher Scientific, Waltham, MA, USA); Hypoxia Chamber (Stemcell Technologies, Vancouver, BC, Canada); and ACQUITY UPLC BEH C18 column (2.1 mm × 100 mm, 1.7 μm, Waters, Milford, MA, USA) were used. Mouse hippocampal neuron cells (HT22 cell) (purchased from Shanghai Kang Lang Biological Technology Co., Ltd., Shanghai, China) were subcloned from HT-4. Calycosin-7-*O*-β-d-glucoside (Chengdu Must Biio-Technology Co., Ltd., Chengdu, China, Lot No. MUST-24042920); 2-Chloro-L-Phenylalanine (Sichuan Weikeqi Biotechnology Co., Ltd., Chengdu, China, Lot No. WP24022203); Edaravone (CSN pharm, North Vancouver, BC, Canada, Lot No. CSN10476-006); Annexin V-FITC/PI Apoptosis Kit (Lianke Bio, Hefei, China, Lot No. A406414); CCK-8 (Glpbio Technology Inc, CA, USA, GK10001); ROS Detection Kit (Abbkine Scientific Co., Ltd., Atlanta, GA, USA, Lot No. ATXC07101); NO Testing Kit (Nanjing Jiancheng Bioengineering Insititute, Nanjing, China, Lot No. 20240910); Glu and GABA Test Kit (The Memphis Institute for Biomedical Innovation & Healthcare Solutions, Memphis, TN, USA, Lot No.S2164N682202L-N1620 and S8630N460035L-N1620) were used.

### 4.2. Methods

#### 4.2.1. Establishment of OGD/R Cell Model

HT22 cells in the logarithmic growth phase were inoculated in 96-well plates at 1 × 10^4^ cells/well and were randomly divided into blank, control, and OGD/R groups. The control group cells were cultured normally, and the cells of the model group were subjected to the OGD/R modeling. HT22 cells were adherently cultured for 24 h, and the original culture medium was discarded. After adding DMEM glucose-free medium (Nanjing SenBeiJia Biological Technology Co., Ltd, Nanjing, China, Lot No. BC20240429), the orifice plate was placed in a hypoxic chamber, and the mixed gas (containing 94%N_2_, 5%CO_2_, 1%O_2_) was introduced into the hypoxic chamber. After 5 min of ventilation, the hypoxic chamber was closed and placed in a 37 °C, 5%CO_2_ constant temperature incubator to continue the culture. The OGD time of the OGD/R group was 2, 4, 6, 8, 10, and 12 h. After the cell treatment, the CCK-8 method was utilized to gauge cell viability. The cell viability was reduced to about 50% as a suitable condition for modeling. The appropriate OGD time was selected.

#### 4.2.2. Determination of the Non-Toxicity Range of CAG and EDA

HT22 was inoculated in 96-well plates and randomly divided into the blank, control, and administration groups. After 24 h of culture, the control group was normally cultured. The drug administration group was added with different concentrations of CAG solution (0, 10, 30, 60, 90, 120, 150, 180, 200 μmol/L) and the positive drug EDA solution (0, 100, 200, 300, 400, 500, 600, 800, 1000 μmol/L), and continued to be cultured for 24 h. The CCK-8 method was utilized to gauge cell viability. We used the dose–response–inhibition module under the GraphPad Prism software (Version 9.0.0) nonlinear regression (curve fit) to fit the cell viability with the logarithmic concentration of CAG, and the maximum half inhibitory concentration (IC50) of CAG was obtained.

#### 4.2.3. Determination of CAG and EDA Administration Dose

HT22 cells were inoculated in 96-well plates and randomly divided into blank, control, OGD/R, and drug-administrated groups. After 24 h of culture, the control group was cultured normally, and both the drug-administrated group and the OGD/R group were modeled using the OGD/R method. After successful modeling, different concentrations of CAG solution (20, 40, 60, 80, 100, 120, 140, 180 μmol/L) and EDA solution (60, 80, 100, 120, 140, 160, 180, 200, 220 μmol/L) were added to the drug-administrated group, and the OGD/R group was added with fresh complete medium (Wuhan Pricella Biotechnology Co., Ltd., Wuhan, China, Lot No. WHAA24K281) and continued to be cultured for 24 h. The CCK-8 method was utilized for gauging cell viability.

#### 4.2.4. Effect of CAG on the Viability of OGD/R-Injured HT22 Cells Detected by CCK-8 Assay

HT22 cells were inoculated in 96 plates at a density of 1 × 10^4^ cells/well, and randomly divided into the blank group, control, OGD/R, EDA, CAG-L, CAG-M, and CAG-H groups. Except for the control group, all the groups were OGD/R modeled, and the administered groups were given a complete medium containing different concentrations of CAG (35, 70, and 140 μmol/L; low-, medium-, and high-dose groups) and EDA (160 μmol/L) after modeling, and the control and OGD/R groups were given complete medium, and the cells were continued to be cultured for 24 h. The cell viability was detected by CCK-8 assay.

#### 4.2.5. Flow Cytometric Detection of the Effect of CAG on ROS Levels in OGD/R-Injured HT22 Cell

Cell grouping, modeling, and drug administration procedures are described under “Section 4.2.4”. HT22 cells were washed 2 times with PBS (Nanjing SenBeiJia Biological Technology Co., Ltd., Nanjing, China, Lot No. BC20240416) after OGD/R modeling and/or drug administration, and PBS was discarded. Trypsin digested the cells, transferred to 1.5 mL EP tubes, and centrifuged at room temperature for 5 min at 3000 r·min^−1^. Serum-free medium was used to formulate the DCFH-DA fluorescent probe so that the final concentration was 10 μmol/L. Serum-free medium was used to wash the cells 2 times, and the cells were counted. After cell counting, cells were resuspended with 1 mL prepared probe solution while avoiding light, with a cell density of 1 × 10^6^ per tube, and incubated in a cell culture incubator at 37 °C for 30 min keeping away from light. The cells were washed with serum-free medium (Wuhan Pricella Biotechnology Co., Ltd., Wuhan, China, Lot No. WHB824D281) 2 times to fully remove the DCFH-DA that had not entered into the cells. The cells were resuspended with 0.5 mL PBS, and the level of ROS was detected by flow cytometry, with 488 nm as the excitation wavelength and 525 nm as the emission wavelength.

#### 4.2.6. Flow Cytometry to Detect the Effect of CAG on the Apoptosis of HT22 Cells Injured by OGD/R

Cell culture, modeling, and drug administration refer to the method under “Section 4.2.4”. The cells were digested with EDTA-free tryptic (Dalian Meilun Biotechnology Co., Ltd. Dalian, China, Lot No. MA0234-1-Mar-27J) digest, and the parameters of the flow cytometer were adjusted according to the instructions in the apoptosis kit. The cells were washed twice by centrifugation with pre-cooled PBS, 1 × 10^6^ cells were collected, and 500 μL of binding buffer was obtained to resuspend the cells. An amount of 5 μL of Annexin V-FITC and 10 μL of PI was added to each tube, incubate for 5 min at room temperature and protect from light to detect apoptosis by flow cytometry.

#### 4.2.7. Colorimetric Measurement of Glu and GABA Concentration and Enzymatic Measurement of NO Concentration in HT22 Cells Supernatant

HT22 cells were inoculated with 1 × 10^5^ cells/well in 24-well plates, and the steps of cell culture, modeling, and drug administration were referred to the method under “Section 4.2.4”. The cell supernatant was collected, and Glu was extracted by homogenization in an ice bath at the volume ratio of 1:5 between cell culture medium and extraction reagent, then centrifuged at room temperature at 10,000 r·min^−1^ for 10 min, and the supernatant was mixed with the color-developing reagent, then a water bath was performed at 90 °C for 20 min, cooled with running water, and 200 μL was placed into a 96-well plate, and the absorbance value was recorded at 570 nm by a microplate reader, and Glu content was calculated according to the standard curve.

The determination of GABA content was based on the reaction of phenol and sodium hypochlorite with GABA to produce blue–green products, which were detected at 640 nm using a microplate reader. An amount of 0.3 mL of supernatant was added with 1 mL of extraction solution, homogenized thoroughly, and then water-bathed at 95 °C for 2 h, cooled down, and then centrifuged at room temperature for 10 min at 10,000 r·min^−1^, and then the supernatant was extracted. The supernatant was mixed with a coloring reagent, and the absorbance value was recorded at 640 nm by a microplate reader, and the GABA content was calculated according to the standard curve.

The nitrate reductase method was used for determination of NO content in cell supernatants. Since the chemical properties of NO are relatively active, it is quickly converted to NO_2_^−1^ in vivo, and NO_2_^−1^ is further converted to NO_3_^−1^. In this study, the nitrate reductase method was used to specifically reduce NO_3_^−1^ to NO_2_^−1^, and the concentration of NO_2_^−1^ was measured at 550 nm by a microplate reader. The absorbance value reflects the NO level. The NO content was calculated according to the instructions of the protocol.

#### 4.2.8. Non-Targeted Metabolomics of CAG-Protected OGD/R-Injured HT22 Cells by UPLC-Q/TOF-MS

Cells were inoculated on 10 cm dishes and randomly divided into the control, OGD/R, EDA, CAG-L, CAG-M, and CAG-H groups, with 7 replicate wells in each group, and referred to “Section 4.2.4” under the treatment of cells. After 24 h of cell administration, 3 × 10^6^ cells were collected from each well, the obtained cells were washed twice with 37 °C PBS, and centrifuged to discard the supernatant, and the samples were added to 800 μL of −20 °C methanol which contained 500 ng·mL^−1^ of 2-chloro-L-phenylalanine as internal standard, vortexed for 1 min, sonicated in an ice bath for 5 min, and centrifuged for 10 min at 4 °C and 12,000 r·min^−1^, and the supernatant was collected in equal volume, transferred to a clean 5 mL glass scale centrifuge tube, and dried by nitrogen gas flow at 40 °C. A total of 100 μL of chromatographic methanol (Merck KGaA, Darmstadt, Germany) was added to the residue, then vortexed for 1 min to re-solubilize the residues, transferred to an EP tube, and centrifuged at 4 °C for 10 min at 12,000 r·min^−1^. Then, the supernatant was placed into an interpolated tube to analyze the sample. All samples were mixed in equal volumes of 5 μL to obtain representative QC samples.

The chromatographic conditions were as follows: ACQUITY UPLC BEH C18 (2.1 mm × 100 mm, 1.7 μm, Waters) column, 0.1% formic acid methanol (A)—0.1% formic acid aqueous (B) as the mobile phase with gradient elution. The following gradient elution procedure was performed: 0–0.5 min, 10–35% A; 0.5–1.5 min, 35–82% A; 1.5–6.5 min, 82–100% A; 6.5–10.5 min, 100–100% A; 10.5–11 min, 100–10% A; 11–13 min, 10–10% A. The injection volume of the positive and negative ion modes was 15 μL, the column temperature was set as 30 °C, the temperature of the sample chamber was all 4 °C, the injector temperature was 4 °C, and the flow rate of the mobile phase was 0.20 mL·min^−1^ (splitless mode).

The mass spectrometry conditions were as follows: electrospray ionization source in positive and negative ion mode scanning, positive and negative mode ion source voltage 5500 V, −4500 V, ion source temperature is 500 °C, the de-clustering voltage ±100 V, the ion source atomizing gas and auxiliary gas pressure are 55 Pa, the curtain gas pressure is 35 Pa. The primary mass spectrometry scanning range of m/z 100–1500 Da uses a collision energy of 45 eV, while the secondary mass spectrometry uses a high sensitivity mode with a range of m/z 50–1500 Da and collision energy of 45 eV. Dynamic background deduction was enabled, and the Analyst TF 1.7 workstation was used for data acquisition, to obtain the data file with .wiff format. The BPC diagram was obtained using Peakview software (Version 2.0.0.7849). The QC samples were repeatedly injected before the formal injection to make the instrument pre-adapted and pre-equilibrated, and the QC samples were interspersed with the samples during the experimental process to investigate the instrument’s stability. One QC sample was injected every 6 samples. In this study, seven samples were collected from each group, and each sample was injected in a single run. The analysis began with a QC sample and ended the entire assay process with an injection of a QC sample. During the detection process, the mass spectrometer was calibrated once for every 7 samples analyzed.

#### 4.2.9. Multivariate Statistical Analysis of Cell Non-Targeted Metabolomics, Differential Metabolite Screening, and Construction of Biological Information Network

The metabolomics data under positive and negative ion modes were obtained. We adopted the Locally Weighted Scatterplot Smoothing (LOWESS) normalization method based on QC samples. The obtained raw data were analyzed for MS-DIAL software (5.1.230912) for peak extraction, peak alignment, and peak filtering processes, and peak identification was accomplished by comparing with public databases. First, a new project was created in the MS-DIAL and LC/MS analysis mode and ion mode were selected, followed by setting the grouping, retention time, and m/z range. Ion peaks with less than 80% probability of occurrence in all groups were removed. QC-4 which resides in the center of the entire injection process was selected for alignment. Firstly, PCA analysis was performed with SIMCA-P 14.1 software. According to the established PCA model, the control, CAG-H, CAG-M, and EDA groups were highly overlapping and significantly different from the OGD/R group in positive and negative ion modes. The results are shown in Figure S3 in the Supplementary Materials. Then, the OPLS-DA model was established successfully. The reliability of the OPLS-DA model was evaluated by seven-fold cross-validation and 200 iteration permutation tests. Taking CAG-H as an example, OPLS-DA models of control vs. model, CAG-H vs. model and EDA vs. model under positive and negative ion modes were constructed. Then, SIMCA-P 14.1 software was used to construct a shared and unique structure plot (SUS plot) based on two OPLS-DA models. VIP > 1, *p* < 0.05, FC > 2 or FC < 0.5, AUC > 0.5 were used as the criteria for screening differential metabolites. S-plot and volcano plots were used to screen differential metabolites. According to m/z, molecular formula, retention time, error/ppm, AUC, and other data of cell metabolites, combined with HMDB (https://hmdb.ca/; accessed on 29 August 2024), Massbank (https://massbank.eu/MassBank/; accessed on 29 August 2024) and PubChem (https://pubchem.ncbi.nlm.nih.gov/; accessed on 29 August 2024) online database, the differential metabolites with significant changes between the cell control group and the OGD/R group were identified. The Wei Sheng xin online platform (http://bioinformatics.com.cn; accessed on 2 January 2025) was used to draw differential metabolites clustering heatmap. CorrelationCalculator (1.0.1) and Cytoscape (3.9.1) software were used to perform the correlation analysis of differential metabolites. Using MetaboAnalyst 6.0 (https://www.metaboanalyst.ca/; accessed on 7 November 2024) online platform for multiple ROC exploratory analysis, with AUC and CI as indicators, to obtain the optimal combination of variables with joint diagnostic value [50]. The Youden Index was calculated by using MedCalc (Version 23.1.3).

The identified differential metabolites were introduced into MetaboAnalyst 6.0 for metabolic pathway analysis. Differential metabolites with significant callback effects by CAG are imported into Cytoscape (3.9.1) software and MetScape plug-in was used to establish a metabolite–gene network and obtain metabolite-related genes. Disease genes were retrieved using ischemic stroke as a keyword through GeneCards (https://www.genecards.org/; accessed on 13 September 2024), DisGeNET (https://www.disgenet.org/; accessed on 13 September 2024), OMIM (https://www.omim.org/; accessed on 13 September 2024), PharmGKB (https://www.pharmgkb.org/; accessed on 13 September 2024), and DrugBank (https://go.drugbank.com/; accessed on 13 September 2024) databases. The Venn diagram captures the intersection of differential metabolite-related genes and disease genes. KEGG enrichment and GO analyses were performed on the selected common gene targets by the Cluego plugin of Cytoscape software. Intersection genes were imported into the STRING online database (https://cn.string-db.org/; accessed on 22 September 2024) for PPI network analysis. The key target protein was obtained with a PPI network degree value greater than or equal to twice the median value in the MCODE subcluster. The joint-pathways analyst module of MetaboAnalyst 6.0 was used to conduct metabolic pathway joint analysis of metabolites and key target proteins. The metabolites were imported into the Pathview online platform (https://pathview.uncc.edu/; accessed on 22 September 2024), which visually displays the metabolite changes in metabolic pathways.

The top-ranked key target protein structure was downloaded from PDB (https://www.rcsb.org/; accessed on 8 October 2024), and the 3D structure of CAG was downloaded from the PubChem database. It was input to the ligand and the receptor Dockthor molecular docking platform (https://dockthor.lncc.br/v2/; accessed on 10 October 2024) for molecular docking. The smaller the binding energy, the more stable the interaction between the component and the target. ChimeraX 1.7.1 software (accessed on 13 October 2024) was used to visualize the docking results, establish the docking interaction model diagram, and then the heatmap of molecular binding energy was drawn.

### 4.3. Data Processing

SPSS 20.0 software was adopted for statistical analysis, and mean ± SD was utilized to express the data. The data met the normality and homogeneity of variance standards, and multiple groups were compared using one-way ANOVA based on the LSD statistical method. If data did not meet normality, then nonparametric tests were used. *p* < 0.05 was defined as a statistically significant difference. Graphs were made with GraphPad Prism 9.0.

## 5. Conclusions

This study aims to deeply explore the protective role of CAG in the process of OGD/R in neuronal cells. We systematically studied the CAG action path and cellular metabolic networks using non-targeted metabolomics techniques and network analysis methods. Studies have shown that CAG can effectively correct the cell metabolic disorders caused by OGD/R injury, especially the abnormal purine metabolism. Its protective mechanism may be related to reducing the oxidative stress state of cells, reducing apoptosis and restoring energy balance. CAG shows good neuroprotective properties, and research suggests that regulating the activity of target enzymes related to purine metabolism may be beneficial for treating ischemic stroke. This study not only helps to reveal the biological activity of CAG but also provides a scientific basis for further development of protective strategies against nerve cell injury.

## Figures and Tables

**Table 1:** OPLS-DA model fitting parameters and CV-ANOVA result.

Ion Mode	R2 (cum)	Q2 (cum)	Source of Error	Sum of Square	Degree of Freedom	Mean Square	F	*p*	Standard Deviation
Positive	0.935	0.522	Total	294.00	294	1.00	1.53	5.60 × 10^−3^	1.00
			Regression	138.29	108	1.28			1.13
			Residual	155.71	186	0.84			0.91
Negative	0.975	0.645	Total	294.00	294	1.00	2.24	5.72 × 10^−7^	1.00
			Regression	184.28	126	1.46			1.21
			Residual	109.72	168	0.65			0.81

**Table 2:** Differential metabolites.

Number	Name	t_R_/min	Measured (m/z)	Theoretical value(m/z)	Error/ppm	Adduct ions	Chemical Formula	MS/MS Fragments	VIP	P_corr_	AUC	Youden Index	FC	OGD/R /Control
1	Xanthosine 5′-phosphate	2.059	363.0323	364.0420	−6.67	[M-H]^−^	C_10_H_13_N_4_O_9_P	78.9562; 96.9694; 108.0213	6.29	−0.65	1.000	1.000	0.004	↓ ***
2	Uric acid	1.506	167.0201	168.0283	−5.89	[M-H]^−^	C_5_H_4_N_4_O_3_	41.9997; 56.0136; 124.6946	1.11	−0.81	1.000	1.000	0.053	↓ ***
3	2-Deoxyribose-5-phosphate	2.326	213.0160	214.0242	−4.62	[M-H]^−^	C_5_H_11_O_7_P	62.9671; 78.9587	2.83	0.74	0.918	0.714	3.529	↑ **
4	Xanthosine	2.648	283.0686	284.0766	0.70	[M-H]^−^	C_10_H_12_N_4_O_6_	80.0249; 108.0227; 151.0287	1.33	−0.82	1.000	1.000	0.061	↓ *
5	Guanosine 5′-monophosphate	2.036	362.0513	363.0580	1.54	[M-H]^−^	C_10_H_14_N_5_O_8_P	78.9600; 133.0275	2.42	−0.70	0.980	0.857	0.060	↓ ***
6	Deoxyguanylic acid	2.541	346.0566	347.0630	2.19	[M-H]^−^	C_10_H_14_N_5_O_7_P	78.9633; 107.0466; 96.9692; 134.0468	1.12	−0.88	1.000	1.000	0.081	↓ ***
7	Folic acid	2.925	440.1311	441.1397	−2.81	[M-H]^−^	C_19_H_19_N_7_O_6_	66.0328; 105.0353; 132.0461	1.17	0.99	1.000	1.000	15.481	↑ ***
8	Uridine 5′-monophosphate	1.893	323.0295	324.0359	2.90	[M-H]^−^	C_9_H_13_N_2_O_9_P	78.9548; 96.9691	1.36	−0.56	1.000	1.000	0.098	↓ **
9	Inosine 5′-monophosphate	2.026	347.0410	348.0470	3.53	[M-H]^−^	C_10_H_13_N_4_O_8_P	65.0510; 78.9575; 94.9416; 135.0486	1.52	−0.66	0.939	0.714	0.109	↓ **
10	Myo-Inositol	1.386	179.0570	180.0634	4.78	[M-H]^−^	C_6_H_12_O_6_	59.0139; 71.0138; 87.0082; 161.0450	1.05	0.64	0.980	0.857	14.032	↑ ***
11	Adenosine Monophosphate	1.987	346.0567	347.0630	2.42	[M-H]^−^	C_10_H_14_N_5_O_7_P	78.9563; 80.8462; 96.9713; 107.0540; 134.0437	2.15	−0.67	0.959	0.857	0.096	↓ **
12	PG38:5	12.451	795.5240	796.5254	7.44	[M-H]^−^	C_44_H_77_O_10_P	59.0168; 78.9866; 96.9812; 152.5184; 171.0132	1.02	−0.55	0.939	0.857	0.153	↓ **
13	Indole-3-acetic acid	3.056	174.0576	175.0633	8.97	[M-H]^−^	C_10_H_9_NO_2_	59.0133; 128.1500; 130.0657	1.81	−0.75	0.980	0.857	0.128	↓ ***
14	1-Dodecanoyl-sn-glycero-3-phosphocholine	8.570	438.2625	439.2700	−0.32	[M-H]^−^	C_20_H_42_NO_7_P	78.9591; 199.1704	1.24	−0.74	0.939	0.857	0.201	↓ ***
15	PG36:3	12.539	771.5184	772.5254	0.36	[M-H]^−^	C_42_H_77_O_10_P	78.9602; 152.9912; 281.2478	1.54	−0.55	0.939	0.857	0.084	↓ ***
16	PG36:4	11.921	769.5031	770.5198	0.76	[M-H]^−^	C_42_H_75_O_10_P	78.9575; 152.9982; 279.2256	1.08	−0.66	1.000	1.000	0.161	↓ **

Note: ↑ indicates upward; ↓ indicates downward; compared with the control group, * *p* < 0.05, ** *p* < 0.01, *** *p* < 0.001 (mean ± SD, *n* = 7).

## Data Availability

Data are contained within the article and Supplementary Materials.

## Supplementary Materials

The following supporting information can be downloaded at: https://www.mdpi.com/article/10.3390/molecules30030549/s1, Figure S1. Chemical structure formula of CAG; Figure S2. Oxygen–glucose deprivation time; Figure S3. PCA diagram; Figure S4. SUS plot; Figure S5. Differential metabolite screening; Figure S6. Metabolite correlation network; Figure S7. Metabolite–gene network and Venn diagram; Figure S8. Potential biomarkers, pathway analysis, and network analysis; Figure S9. Heatmap and visualization of molecular docking binding energy; Figure S10. Visualization of molecular docking of EDA and Adk.
