# Supplementary material for: The Study of the Protection Mechanism of Calycosin-7-O-β-d-Glucoside Against Oxygen–Glucose Deprivation/Reperfusion in HT22 Cells Based on Non-Targeted Metabolomics and Network Analysis
Source: Molecules. 2025 Jan 25;30(3):549. doi: 10.3390/molecules30030549 (PMC11819903; doi:10.3390/molecules30030549)
Supplement: Supplementary file 1 [file molecules-30-00549-s001.zip › molecules-3370844-supplementary.pdf]

Supplementary material

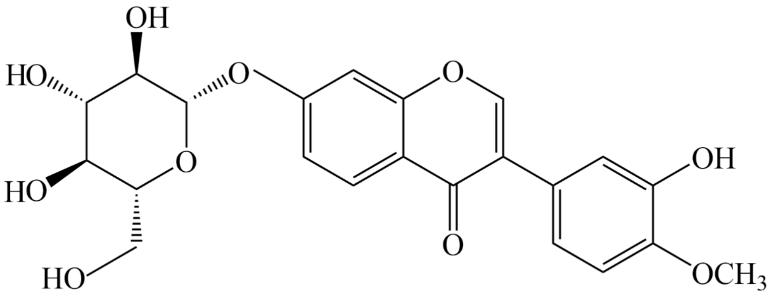

Figure S1. Chemical structure formula of CAG.

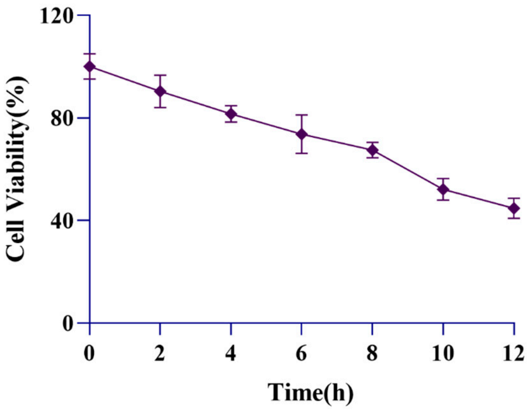

Figure S2. Oxygen-glucose deprivation time (mean±SD, n=6).

According to the established PCA model, the control, CAG-H, CAG-M, and EDA groups are highly overlapping and significantly different from the OGD/R group in positive and negative ion modes (See Figure S3). To search for differential metabolites, we used the OPLS-DA model for further analysis.

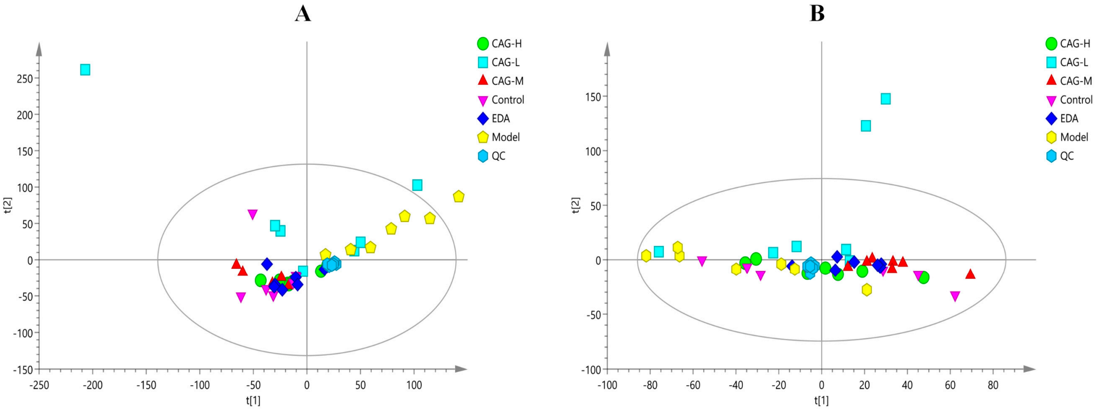

Figure S3. PCA diagram; A: Positive ion mode; B: Negative ion mode.

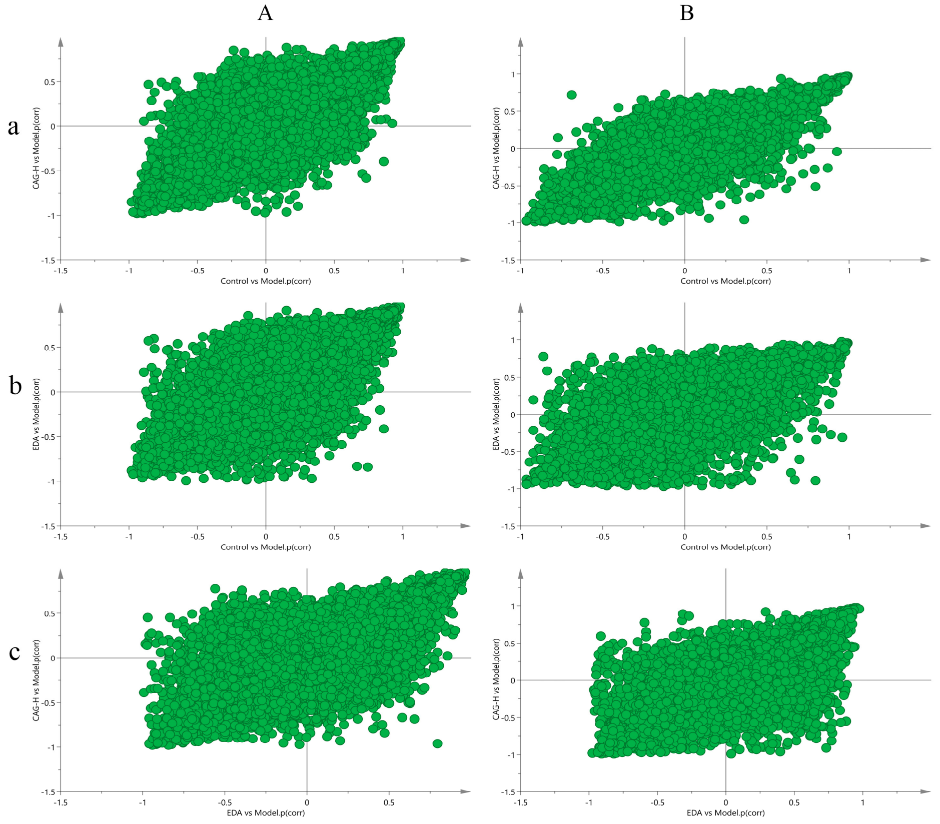

Figure S4. SUS-plot. A: Positive ion mode; B: Negative ion mode; a: Control *vs* model-CAG-H *vs* model; b: Control *vs* model-EDA *vs* model; c: EDA *vs* model-CAG-H *vs* model.

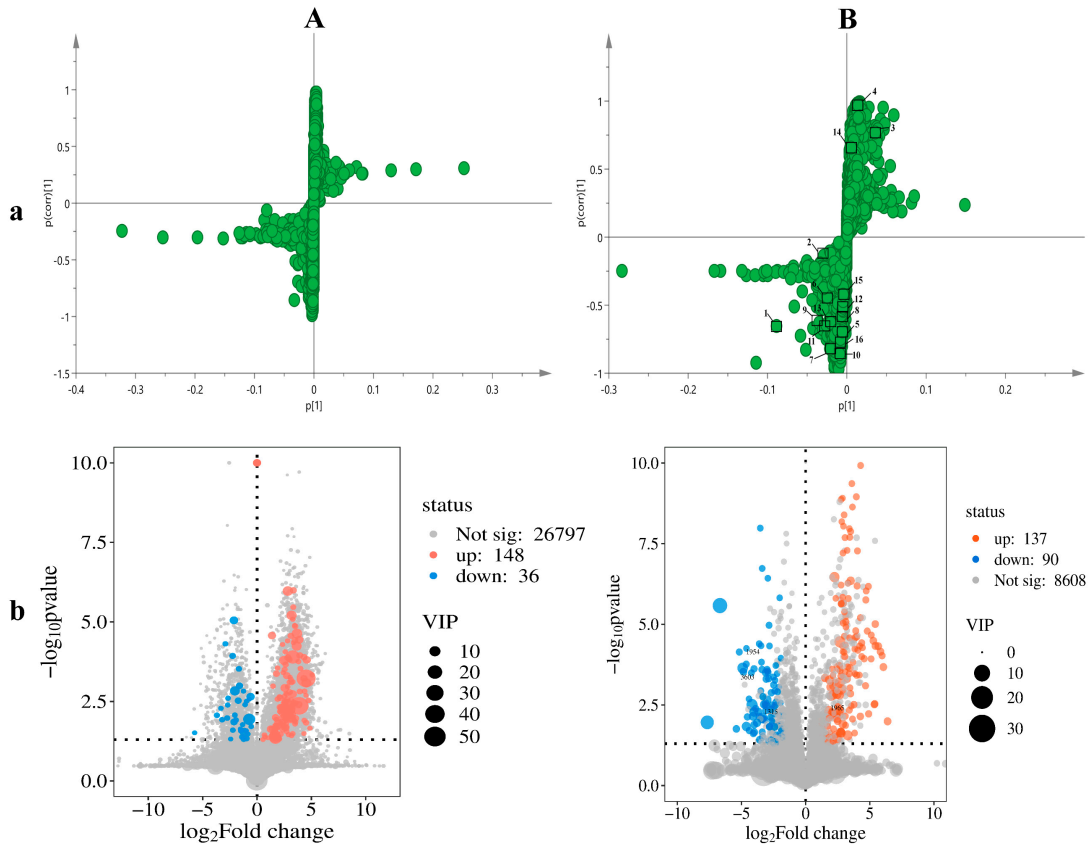

Figure S5. Differential metabolite screening. A: Positive ion mode; B: Negative ion mode; a: S-plot. In B-a, from 1 to 16 are Xanthosine 5'-phosphate, Uric acid, 2-Deoxyribose-5-phosphate, Folic acid, 1-Dodecanoyl-sn-glycero-3-phosphocholine, PG 36:3, Xanthosine, PG 36:4, Guanosine 5'-monophosphate, Deoxyguanylic acid, Adenosine Monophosphate, Uridine 5'-monophosphate, Inosine 5'-monophosphate, Myo-Inositol, PG 38:5, Indole-3-acetic acid. b: Volcano plots based on VIP.

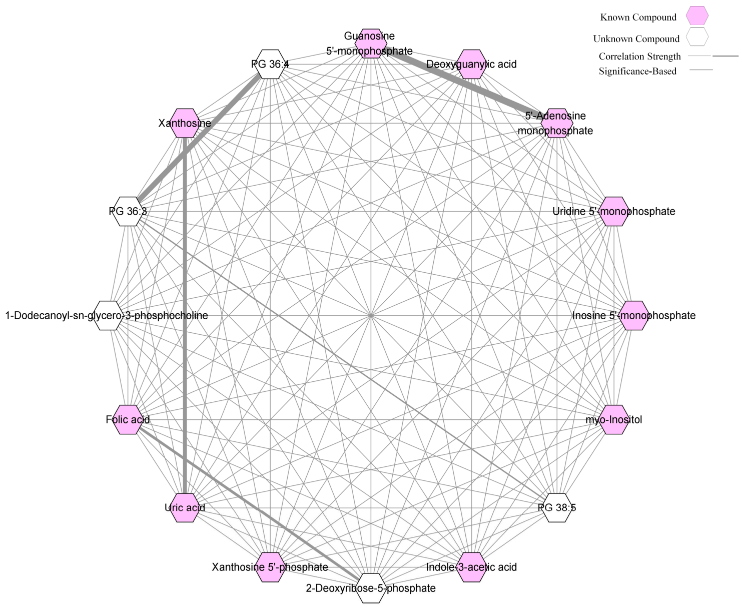

Figure S6. Metabolite correlation network.

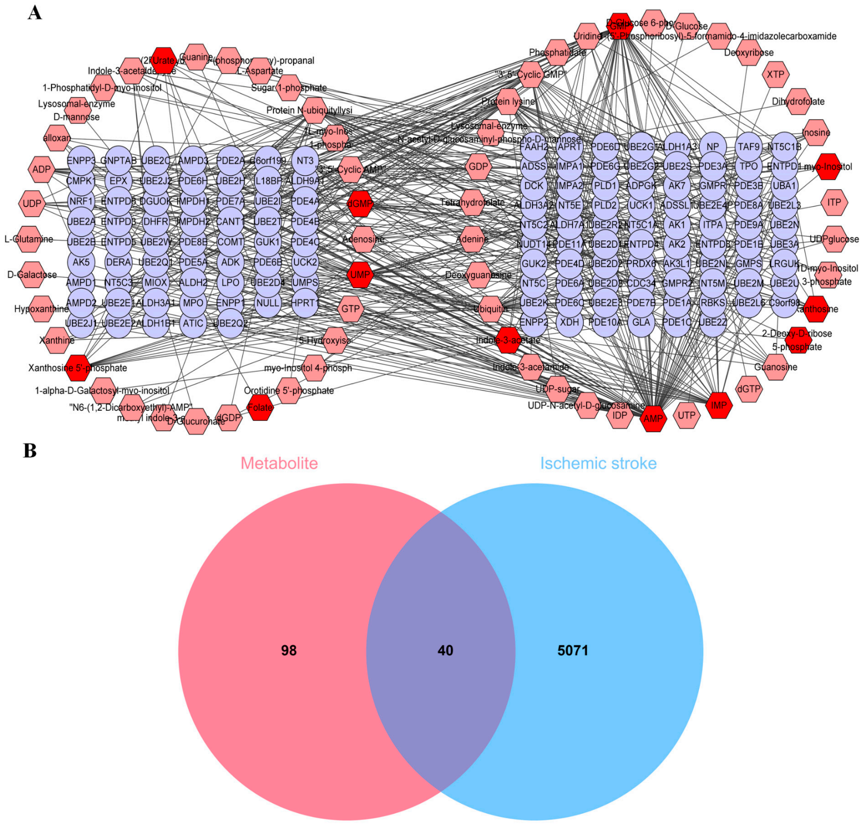

Figure S7. A: Metabolite-gene network; B: Venn diagram of metabolite-related genes and disease genes.

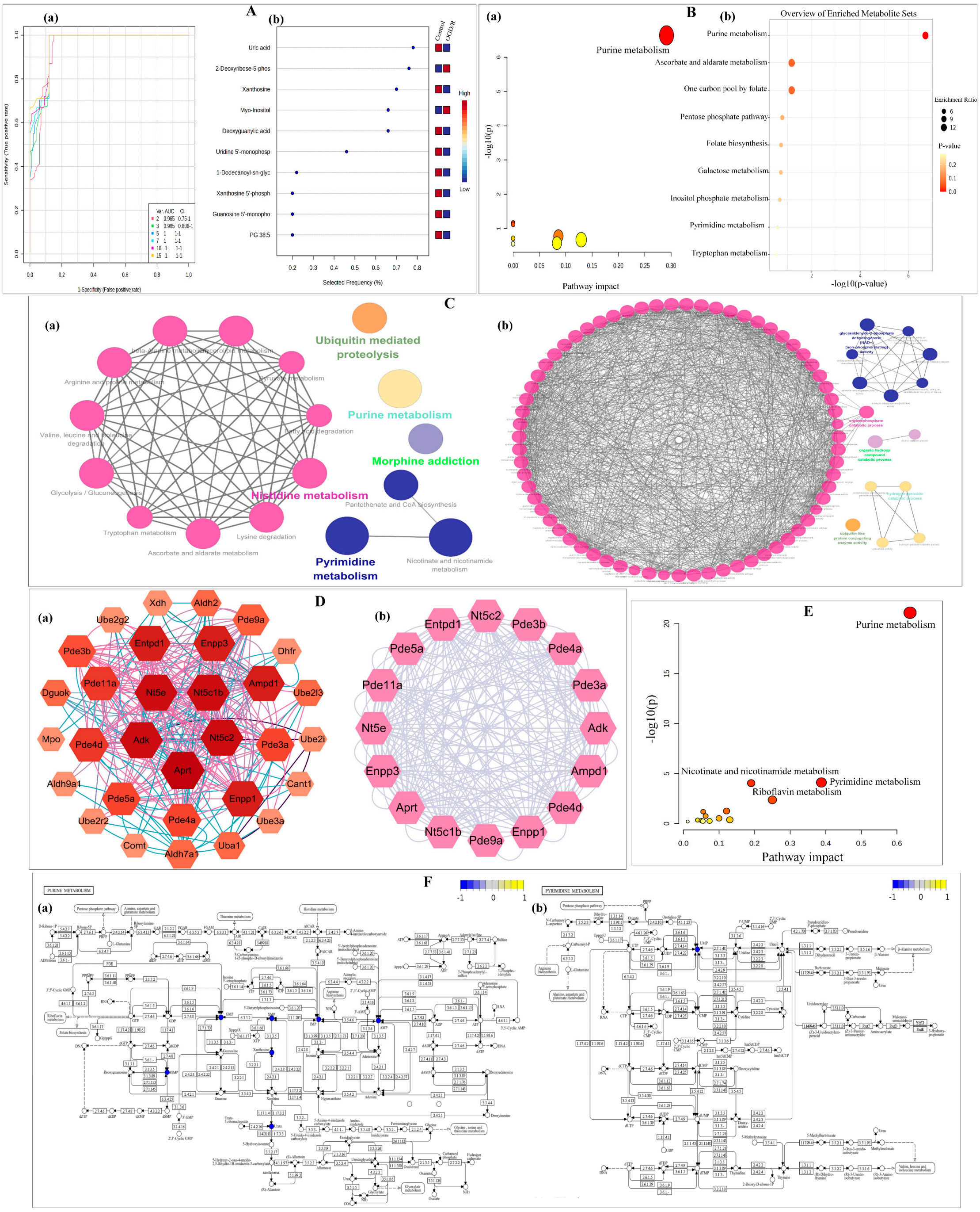

Figure S8. Potential biomarkers, pathway analysis and network analysis. A: Multivariate ROC exploratory analysis. A-a: ROC curves containing different combinations of differential metabolites with corresponding AUC values and confidence intervals (AUC is the area under the curve of different combinations of differential metabolites, Var denotes the number of differential metabolites in the optimal combination, and CI is the 95% confidence interval. This study showed that based on the AUC and CI, the system recommended Var (5) as the best combination model). A-b: The top 10 ranked important potential biomarkers based on the frequencies selected during the cross-validation process, where Uric acid, 2-Deoxyribose-5-phosphate, Xanthosine, Myo-Inositol and Deoxyguanylic acid are the best metabolite combinations for differentiating the model cells from normal cells). B: Metabolic pathway analysis. B-a: Pathway analysis; B-b: Enrichment analysis. Purine metabolism is a metabolic pathway that meets both $P < 0.001$ and impact value > 0.2. C: KEGG and GO analytics for network construction. C-a: KEGG enrichment analysis; C-b: GO analysis. D: D-a: Protein-

protein interaction network (Node size and color depth indicate the degree of the node, that is, the number of interactions that the protein has with other proteins. The larger the node, the darker the color, the more interaction that protein has with other proteins); D-b: Key protein network. E: Joint-pathway analysis of key proteins and differential metabolite. The pathways with $P < 0.05$ and impact value > 0.1 were purine metabolism, pyrimidine metabolism, nicotinate and nicotinamide metabolism, and riboflavin metabolism. F: Metabolic pathway trend diagram. F-a: Purine metabolism trend diagram; F-b: Pyrimidine metabolism trend diagram.

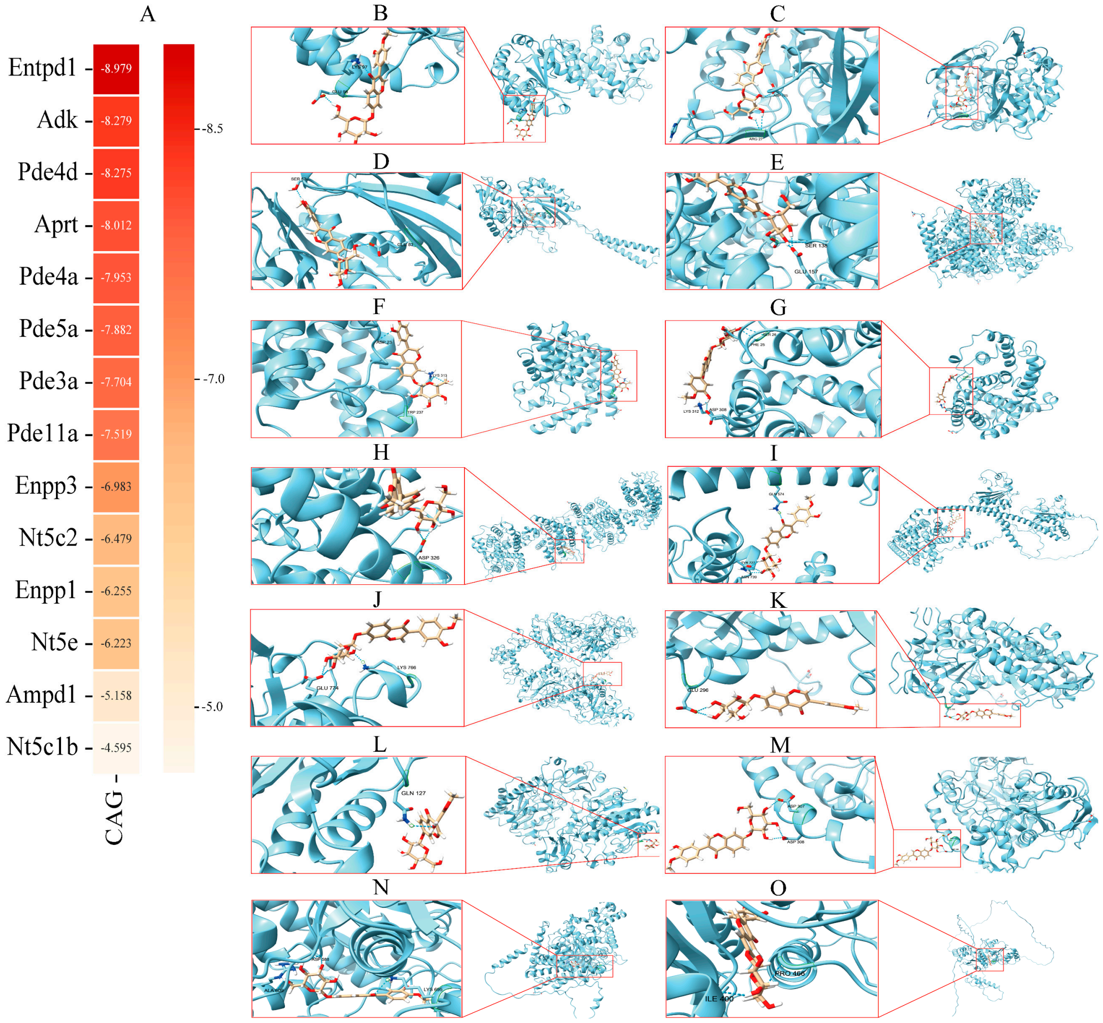

Figure S9. Heatmap and visualization of molecular docking binding energy. A: Molecular binding energy heatmap; B-O is the visualization diagram of the interaction between key proteins and CAG, namely Adk, Aprt, Entpd1, Pde4d, Pde4a, Pde5a, Pde3a, Pde11a, Enpp3, Nt5c2, Enpp1, Nt5e, Ampd1, Nt5c1b.

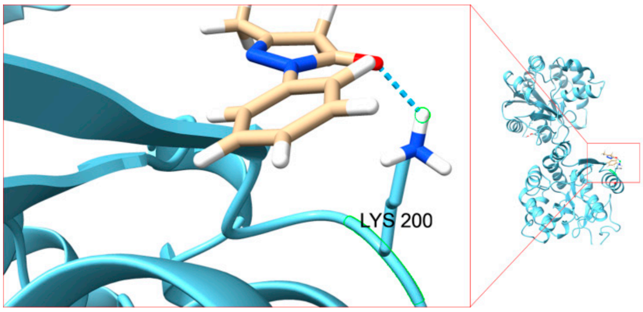

Figure S10. Visualization of molecular docking of EDA and Adk with the binding energy of -6.933 kcal·mol^{-1}.
